# Poisoning by Strychnine

**Published:** 1855-01

**Authors:** J. J. Lescher

**Affiliations:** Mount Carmel, Ill.


POISONING BY STRYCHNIA. By J. J. LESCHER, M. D., of Mount Carmel, Ill.—On the morning of the 19th of October, my brother, Dr. Geo. Lescher, was requested to visit E. W., a German laborer, aged 30 years, of a powerful frame, at the "Lock of the Grand Rapids," situated on the Indiana bank of the river, two miles from this place. The messenger represented the patient as having been seized violently with a "congestive chill." When my brother reached the "Lock," he found the patient very ill, though not in a condition such as is usually met with in persons suffering from the cold stage of a malignant intermittent. However, as the patient's extremities had been wrapped up in strong mustard cataplasms before my brother saw him, he regarded the singularity of the symptoms as indicative of a partial reaction, and accordingly directed his efforts to establish it fully. As the patient was exceedingly restless, manifesting a constant disposition to convulsions, my brother administered nothing but opiates and brandy, which did not seem to control the tremours in the slightest degree. At the expiration of an hour, a violent convulsion came on, affecting the entire frame, which it was supposed would terminate fatally. I was immediately sent for, and saw him about three and a half hours after he was taken with what they termed a "fit."

When I first saw him, he was lying on his back, with his upper and lower extremities extended and *rigid;* his arms and legs cold; face flushed, and bathed with cold and clammy perspiration; eyes highly injected, and in constant motion, as from seemingly great affright—as, indeed, his mind fully evidenced; pupils contracted; pulse 120, small, regular and *corded* or *tense* and *strong.* He was making constant motions with his lips, as though he desired drink.

His answers to my inquiries were rapid and unconnected, from which, in connection with the rigid state of all the voluntary muscles, and a seeming tendency to episthotonos, I was led to suspect a serious lesion of the brain and spinal marrow—a condition of cerebro-spinal congestion. Having repeatedly treated the patient for serious and grave attacks of intermittent, and taking into consideration the malarious locality in which he then was, I made but few inquiries in regard to the present attack. His employer not being able to give me any definite information relative to the invasion of what *he* regarded as an aggravated intermittent, under which he had repeatedly seen him suffer, I was thrown off my guard respecting the *tetanic* condition of the muscular system, though I was struck with the *anomalous* symptoms present in the case. I immediately corded the arm; the movement in doing this threw him into a frightful spasm, causing him to elevate his body, whilst he seemed to rest on his heels and the back of his head. As soon as he became somewhat quiet, I abstracted thirty-two ounces of blood. Whilst the blood was flowing, his pulse lessened in frequency and increased in volume. He seemed more quiet, though every attempt to move *threatened a convulsion.* I then gave him—S. quinia gr. xii, et hydrarg. sub. mur. ℈j, mixed with syrup, which he swallowed with *extreme difficulty.* In half an hour he took a like quantity of s. quinia, joined with cal. ℈ss, et s. morph. gr. $\frac{1}{2}$.

I left him then in charge of my brother, and went to the house of the patient's employer, and expressed my doubts in reference to the nature of the patient's disease. He asked me whether the cause of the ailment might be poison. I replied that the symptoms pointed to such a cause; though I supposed, from all the facts which I had elicited, it could not be possible. He then reminded me that he had purchased six grains of strychnia of my partner, Dr. Miller, only a few days before. I asked him what became of it. He said he had placed it on the *mantel-piece* in his dining-room, but, upon looking for it, he could not find it.

My suspicions were at once aroused. I was told that the patient, after eating a *hearty* breakfast, took out of his pocket, in the presence of the family, a paper containing what he supposed to be quinia, and poured a part of the contents into a tumbler containing half a gill of water, and drank it; as part of the powder adhered to the bottom, he added some more water to it, and swallowed what remained. Fifteen minutes after, whilst milking, he fell backwards in a "fit," in which state they conveyed him to the "Lock House."

Upon receiving this clue of the case, I went to the patient, and, upon inquiry, ascertained that he had placed the remainder of the powder into his pocket; and, on searching, found a paper marked "Poison" in his coat-pocket. He assured me that he had taken the "quinia" out of the paper so marked, and observed that he had been taking it for some days past in order to avoid a return of ague. He persisted that he had found it on a shelf in his sleeping-room, and believed it quinia, though its taste differed from any he had used previously. We, however, believed that he had taken it from where his employer had placed it, from motives of *economy*. The family are in the constant habit of keeping quinia about the house, which the laborers knew; and hence E. W.'s filching, under the impression that he would save the expense of a dose of quinia. Upon weighing the remnant of the powder, there were but two grains left, the patient having taken four grains.

Four hours had now elapsed since swallowing the poison, and the patient had not vomited. I made a mixture of warm water and flour, and, with great difficulty, *drenched* the patient, at short intervals, with about three pints of it, alternating it with a solution of tartar emetic. We next made him swallow the whites of about half a dozen eggs, when free emesis followed. I then left him, with directions that he should continue to take the whites of eggs freely, and also use injections. During the night he was purged copiously, and on the *third* day succeeding the poisoning he came to town, and has been quite well up to this period.

